# Effect of Cigarette Smoking on Hearing Levels in Young and Middle-Aged Males

**DOI:** 10.7759/cureus.15093

**Published:** 2021-05-18

**Authors:** Asghar Hussain Syed, FNU Hina, Aakash Chandnani, Vikash Kumar, Jitesh Kumar, Ishan Garg, Faryal Anees, Simra Shahid, Dua Khalid, Besham Kumar

**Affiliations:** 1 Internal Medicine, Royal College of Physician, London, GBR; 2 Cardiology, National Institute of Cardiovascular Diseases, Karachi, PAK; 3 Family Medicine, Ziauddin University, Karachi, PAK; 4 Internal Medicine, Liaquat University of Medical and Health Sciences, Jamshoro, PAK; 5 Internal Medicine, Jinnah Sindh Medical University, Karachi, PAK; 6 Neurology, Ghulam Muhammad Mahar Medical College, Sukkur, PAK; 7 Internal Medicine, Ghulam Muhammad Mahar Medical College, Sukkur, PAK; 8 Clinical Medicine, Ross University School of Medicine, Bridgetown, BRB; 9 Medicine, Jinnah Sindh Medical University, Karachi, PAK; 10 Internal Medicine, Jinnah Postgraduate Medical Centre, Karachi, PAK

**Keywords:** hearing, decibel, smoking, risk factor, association

## Abstract

Introduction: Smoking is a well-recognized risk factor for many health issues; however, its association with hearing loss has been a debate. Some studies have shown a positive association while others did not. In this study, we aim to identify the effect of cigarette smoking on hearing in our population.

Methods: This cross-sectional study was conducted in a tertiary care hospital in Pakistan from August 2020 to March 2021. Five hundred male smokers (n = 500), with a history of smoking for more than three years between the ages of 21 and 50, were enrolled in the study via consecutive convenient non-probability sampling after informed consent. Five hundred male non-smokers (n = 500) were enrolled as a reference group. Audiometry was performed in a soundproof room.

Results: The hearing levels in audiometry were significantly higher in smokers compared to non-smokers (22.8 ± 8.12 decibels vs 18.7 ± 6.12; p-value < 0.0001). Participants who had been smoking for more than 10 years had higher hearing levels in the audiometry test compared to the participants with less than 10 years of smoking history (24.21 ± 8.91 decibels vs. 21.1 ± 8.01 decibels: p-value < 0.0001).

Conclusion: In this study, smokers were associated with greater loss in hearing compared to non-smokers. In addition to other adverse events associated with smoking, smokers should be counselled about hearing loss related to it.

## Introduction

Among the five basic senses humans have, hearing is one of the most important tools for social connectivity and communication. As per World Health Organization (WHO), it has been found that hearing loss is one of the greatest causes of human disability with a prevalence of over 5% of the worlds’ population, approximating up to 250 million people [1,2]. Disabling hearing loss can be defined as an average hearing threshold of 40 dB hearing loss. The auditory function is known to deteriorate gradually during the ageing process however there are certain other medical and environmental factors like hypertension, diabetes, chronic ear infections, use of ototoxic medications and alcohol, smoking, occupational or leisure noise exposure that increases the disease burden [3-5].

Smoking is a well-recognized risk factor for many health issues, such as lung cancer and cardiovascular diseases; however, its association with hearing loss has been a debate. Some studies have shown a positive association while others did not. Several theories have been put forth to explain the effect of nicotine on the auditory system, including vasospasm of the small blood vessels of the inner ear, ototoxicity, increased blood viscosity and cochlear ischemia [1,4]. Passive smoking is related to mild sensorineural hearing loss [3]. Since older people have mostly smoked cigarettes for a longer period than their young counterparts, the incidence of impaired hearing is more frequent in the 30s and 40s age group [1]. Other studies further examined the relationship between smoking and noise-induced hearing loss and have found synergistic influence over each other [2,5]. Others have suggested that not only heavy but light smoking is also toxic and can result in the development of hearing loss [6].

There is very limited data available that study the impact of smoking on the hearing level. In this study, we aim to identify the effect of cigarette smoking on hearing in our population. By identifying smoking as a risk factor for hearing loss, we can counsel patients about the implication of smoking on hearing.

## Materials and methods

This cross-sectional study was conducted in a tertiary care hospital in Pakistan from August 2020 to March 2021. Five hundred male smokers (n = 500), with a history of smoking for more than three years between the ages of 21 and 50, were enrolled in the study via consecutive convenient non-probability sampling after informed consent. Five hundred male non-smokers (n = 500) were enrolled as a reference group. Participants with age more than 50 years, pre-existing ear deformity, hypertension, diabetes and patients on ototoxic drugs were excluded from the study, as all of them are known risk factor for hearing loss. Ethical review board approval was taken before enrolment of the patients.

Patients’ characteristics including age, duration of smoking, body mass index (BMI), systolic blood pressure (SBP), and diastolic blood pressure (DBP) were noted in a self-structured questionnaire. The audiometry was performed in a soundproof room. Trained otolaryngologist technicians performed tests and collected data at different frequencies for each ear at six different frequencies, i.e. 0.5, 1.0, 2.0, 3.0, 4.0, and 6.0 kilohertz (kHz). The final hearing level was taken as the mean of hearing levels of both ears.

Statistical analysis was done using Statistical Package for Social Sciences® software version 23.0 (SPSS; IBM Corp., Armonk, NY, USA). Categorical data such as age distribution was presented as frequency and percentage. Numerical data such as BMI and hearing level were presented as mean and standard deviation. t-Test and Chi-square were applied as appropriate. A p-value of less than 0.05 meant that the difference between the groups is significant and the null hypothesis is void.

## Results

The distribution of age group, BMI, SBP, and DBP was comparable between the two groups (Table 1).

**Table 1:** Demographics of the participants BMI: body mass index, SBP: systolic blood pressure, DBP: diastolic blood pressure, kg: kilogram, m: meter, mmHg: millimetre mercury

Characteristics	Smoker (n = 500)	Non-smoker (n = 500)	P-value
Age Group			
21 to 30	141 (28.2%)	159 (31.3%)	0.46
31 to 40	201 (40.2%)	191 (38.2%)	
41 to 50	158 (31.6%)	150 (30.0%)	
Measurements			
BMI (kg/m^2^)	22.8 ± 3.1	22.5 ± 3.2	0.13
SBP (mmHg)	106.22 ± 9.2	105.12 ± 9.0	0.05
DBP (mmHg)	75.21 ± 6.1	75.31 ± 6.0	0.79

The hearing levels in audiometry were significantly higher in smokers compared to non-smokers (22.8 ± 8.12 decibels vs 18.7 ± 6.12; p-value < 0.0001) (Table 2).

**Table 2:** Comparison of hearing levels between smokers and non-smokers dB: decibels

Hearing levels (dB)	Smoker (n = 500)	Non-smoker (n = 500)	P-value
	22.8±8.12	18.7± 6.12	< 0.0001

Duration of smoking also affected the hearing levels. Participants who had been smoking for more than 10 years had higher hearing levels in audiometry test compared to participants with less than 10 years of smoking history (24.21 ± 8.91 decibels vs. 21.1 ± 8.01 decibels: p-value < 0.0001) (Table 3).

**Table 3:** Correlation of duration of smoking with hearing loss dB: Decibels

Duration of smoking	Frequency (percentage)	Hearing levels (dB)	P-value
Less than 10 years	261 (52.2%)	21.1 ± 8.01	< 0.0001
More than 10 years	239 (47.8%)	24.21±8.91	

## Discussion

The result of our study highlights the effect of cigarette smoking on hearing levels. Moreover, higher audiometry tests were observed in participants having a smoking history of more than 10 years compared to the ones of less than 10 years. Hence, indicating that duration of smoking has a major impact on hearing levels.

Several studies have been conducted to demonstrate the association of cigarette smoking with hearing loss. Our findings are in line with the findings of a large-scale cohort study conducted among a Japanese working population, which also demonstrated the association between smoking and hearing loss [7]. Similarly, the findings of a meta-analysis conducted by Nomura et al. also showed the same results [1]. However, the results of our study showed diversity with some of the cohort studies which might be due to the methodological differences [8-11], but some of them are consistent with our results [12,13]. The mechanism of smoking's effect on hearing loss is unclear. Some of the risk factors, including elevated blood pressure, hypertension, diabetes mellitus and elevated cholesterol play an important role in the pathogenesis of hearing loss by affecting the cochlea via vasoconstriction. Moreover, due to the vascular nature of stria vascularis, vasocontriction causes by these factors make it more vulnerable to vascular compromise, eventually leading to hearing loss [14]. Hearing loss is of great importance in public health. It has a major impact on social interaction and quality of life. Previous interventions and treatment of the above-mentioned risk factors cause a great decline in their incidence, but further methods are needed to treat them [15]. There were few limitations to our study as well. First, since it was a single-centre study, the sample size was less diverse. Second, because of its cross-sectional nature, the definite association between smoking and hearing loss cannot be established. Third, only male participants were included in the study, as due to the taboo associated with it, female smokers were hesitant to be part of this study. Another limitation was that hearing loss was not stratified according to the number of cigarettes per day due to a lack of available data.

The scientific rationale of this study is to provide strong evidence to support that smoking is a risk factor for hearing loss. It further emphasizes the need for smoking cessation to delay the developments of hearing loss. Moreover, it highlights the need for counselling or help from a doctor or therapist. This type of counselling may include information about the effects of smoking on the body, advice on ways to quit, and other assistance that may improve the chances of quitting. Awareness of triggers for smoking can be gained in therapy and alternative behaviours can be explored, both of which may help break the habit of smoking. It further helps to prevent its complications and to improve the quality of life.

## Conclusions

In this study, smoking was associated with increased hearing loss in young and middle-aged males. Hearing loss was also directly proportional to the duration of smoking. In addition to other adverse events associated with smoking, smokers should be counselled about hearing loss related to it. Smokers should have their hearing tested frequently for timely intervention. Counselling about the adverse event of smoking may help prevent hearing loss in smokers.
